# Specificity profiling of CRISPR system reveals greatly enhanced off-target gene editing

**DOI:** 10.1038/s41598-020-58627-x

**Published:** 2020-02-10

**Authors:** Yao Wang, Mingrui Wang, Ting Zheng, Yingzi Hou, Pingjing Zhang, Tao Tang, Jing Wei, Quan Du

**Affiliations:** 1 State Key Laboratory of Natural and Biomimetic Drugs, School of Pharmaceutical Sciences, Peking University, Beijing, 100191 China; 2 Biomics Biotechnologies co. Ltd, Nantong, 226016 Jiangsu Province China; 3 Department of Obstetrics & Gynaecology, Faculty of Medicine, The Chinese University of Hong Kong, Shatin, New Territories, Hong Kong China; 4 Department of Gynecology, Pan Yu Central Hospital, Guangzhou, China; 5 The Sixth Medical Center of Chinese PLA General Hospital, Beijing, 100048 China; 6 Cancer Research Center Nantong, Tumor Hospital Affiliated to Nantong University, Nantong, 226361 Jiangsu PR China

**Keywords:** Targeted gene repair, Targeted gene repair, Double-strand DNA breaks, Double-strand DNA breaks, Molecular medicine

## Abstract

To explore the editing specificity of CRISPR/Cpf1 system, effects of target mutation were systematically examined using a reporter activation assay, with a set of single-nucleotide mutated target site. Consistent with our previous study performed with CRISPR/Cas9, a “core” sequence region that is highly sensitive to target mutation was characterized. The region is of 4-nucleotide long, located from +4 to +7 position of the target site, and positioned within a positively charged central channel when assembled into Cpf1 endonuclease. Single-nucleotide mutation at the core sequence could abolish gene editing mediated by a however active sgRNA. With a great majority of the target sites, a kind of ‘super’ off-target gene editing was observed with both CRISPR/Cpf1 and CRISPR/Cas9. For a given target site, mutation at certain positions led to greatly enhanced off-target gene editing efficacy, even up to 10-fold of that of the fully-matched target. Study further found that these effects were determined by the identity of target nucleotide, rather than the nucleotide of crRNA. This likely suggests that the interactions between target nucleotide and the endonuclease are involved in this process.

## Introduction

As ancient acquired immune systems of bacteria and archaea, CRISPR (clustered regularly interspaced short palindromic repeat) systems enable also effective gene editing in mammalian cells, and thus have been implemented in basic and clinical relevant studies^1,2^. In the case of a Type II CRISPR system, a Cas9 endonuclease complex with two small RNA components, a CRISPR RNA (crRNA) and a trans-activating crRNA (tracrRNA), to form a sequence-specific CRISPR/Cas9 complex. Targeting specificity of the complex can be easily reprogrammed by modifying the sequence of crRNA^3,4^. Gene editing of the complex generates a sequence-specific and double-strand break (DSB) of chromosomal DNAs. By means of homologous recombination or non-homologous end joining, the double-stranded cleavages will be repaired and thus results in gene-specific modification. Due to its high specificity, efficacy and feasibility, CRISPR/Cas9 has become the most applied genome editing technology. However shortly after its development, off-target gene editing was reported^5,6^. As a major concern of this kind of technology, off-target editing can cause permanent sequence alteration, which leads to genome instability or disruption of normal gene function.

To explore the editing specificity of CRISPR/Cas9, a highly sensitive assay was performed in our previous study^7^. With all possible single-nucleotide mutated targets, wide-spread off-target gene editing was found with all the tested sgRNAs. In addition to the specificity profiles, a “core” sequence region that is highly sensitive to target mutation was identified, locating a few bases upstream of PAM region. Sequence mutation at the “core” region abolishes gene editing by a active sgRNA. However for several target sites, single-nucleotide mutation at certain positions led to greatly enhanced off-target gene editing, even higher than that of the fully-matched target. Taking these general and specific off-target gene editing together, serious safety concerns were raised.

As a more recently developed Type II CRISPR system, CRISPR/Cpf1 was proposed to have a stricter targeting specificity than CRISPR/Cas9^8,9^. To examine this speculation, gene editing specificity of CRISPR/Cpf1 was investigated in the present study, by means of a similar approach as did with CRISPR/Cas9.

## Results

**Figure 1:**
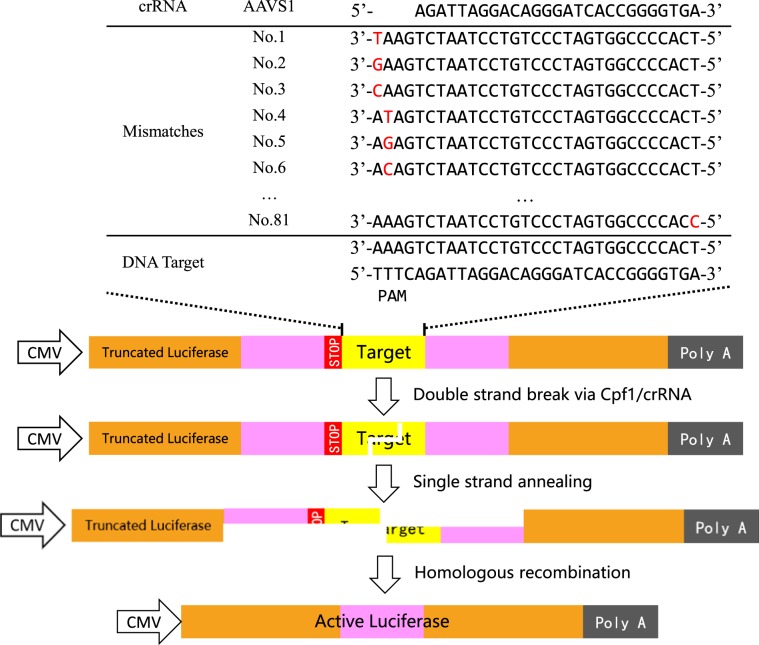
The schematic to examine gene editing specificity of a crRNA. For a given crRNA, a set of target sequences carrying all possible single-nucleotide mutation was examined, by means of a reporter activation assay.

To explore gene editing specificity of CRISPR/Cpf1, a modified reporter activation assay was developed. In addition to a reference reporter gene, three other components are engaged in the assay, including a Cpf1 endonuclease, a crRNA and a target DNA. Cpf1 endonuclease is encoded by pCpf1 vector, crRNA is encoded by pcrRNA vector, target sequence within a split *Firefly* luciferase gene is encoded by pTarget vector, reference gene *Renilla* luciferase is encoded by pRL-TK vector. Serving as a target of gene editing, coding sequence of *Firefly* luciferase is split into two parts by the target sequence of a crRNA, rendering the reporter inactive. At their adjacent ends, each contains an identical sequence of 1000 bp. This allows for a homologous recombination after Cpf1 cleavage, and generates an active *Firefly* luciferase gene (Fig. 1). Therefore, activation of *Firefly* luciferase gene will occur after target editing and homologous recombination. In relative to the originally inactive luciferase, activation of the reporter is quantitatively determined by examining the increase of luminescence in CRISPR/Cpf1-treated samples.

### Specificity profile of CRISPR/Cpf1

Using this sensitive assay, gene editing efficacy of three active crRNAs was examined. In order to make the study comparable to that of Cas9, the three crRNAs were selected from our earlier study with CRISPR/Cas9^7^. For a given crRNA, single-nucleotide mutations were made at every position of the target site. Mutations were also made at PAM region, as well as the adjacent positions downstream of PAM (Fig. 1). Therefore for each crRNA, 81 synthesized DNA fragments carrying the fully matched or single-nucleotide mutated targets were cloned into pTarget plasmid, generating a library of single-nucleotide mutated targets. The recombinant vectors were transfected individually into HEK293 cells, together with pCpf1, pcrRNA and reference vector. Two days after transfection, activation of *Firefly* luciferase was quantitatively determined using dual-luciferase reporter assay.

**Figure 2:**
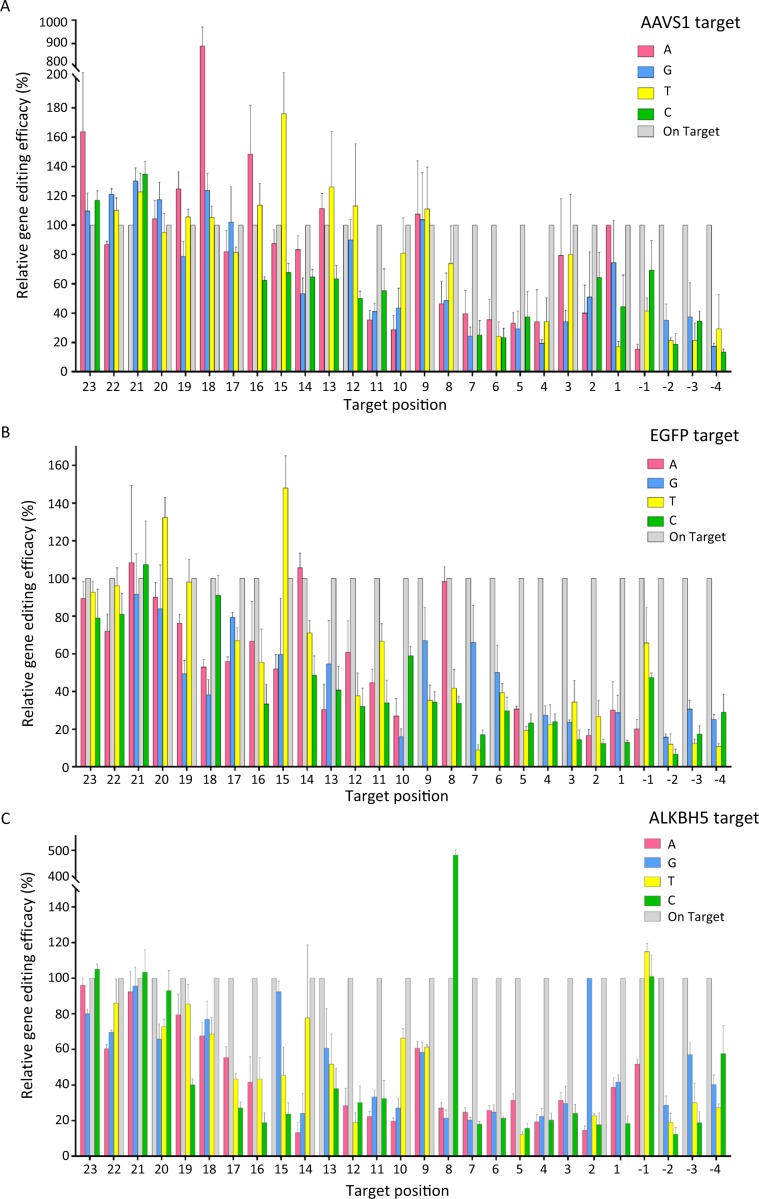
Gene editing specificity across the whole target site by CRISPR/Cpf1 system. Gene editing efficacies of a crRNA were examined with cultured HEK293 cells, and plotted across the whole target site in terms of the identity and the position of the nucleotide mutated. Target positions were numbered in the 5′ to 3′ direction. PAM sequences were numbered as position −1, −2, −3 and −4. Horizontal axis, target site of a crRNA; vertical axis, gene editing efficacy. White columns, the normalized gene editing efficacy of the fully matched sequence. A reference site with scrambled sequence was included. (**A**) Relative gene editing efficacy of *AAVS1* target. Reference gene editing efficacy of the negative control is 19.3%. (**B**) Relative gene editing efficacy of *EGFP* target. Reference gene editing efficacy of the negative control is 17.3%. (**C**) Relative gene editing efficacy of *ALKBH5* target. Reference gene editing efficacy is 18.4%. The data were average values of three biological replicates, all the assay was performed in triplicates.

With a reference of the fully-matched target, all the tested crRNAs mediated efficient gene editing, resulting in a boost of luciferase activity up to 11-fold. To facilitate data analysis, editing efficacy of the fully-matched target was set as 100%, for each crRNA. Then, the relative gene editing efficacy was determined for each single-nucleotide mutated target (Fig. 2). Positions across the target sequence were numbered as position (+1) to position (+23), starting from the 3′ end of PMA region. PAM positions were accordingly numbered as position (−4) to position (−1). Background luciferase activities were determined with scrambled target sequences.

In the process of CRISPR-mediated gene editing, PAM recognization is a critical step^10^. In agreement with the dominant PAM sequence of TTTN^11^, mutations at position (−1) were partly tolerated, while the mutations at position (−2), (−3) and (−4) were not tolerated at all, leading to greatly decreased editing efficacies. For mutations made on the target sequence, editing efficacies were found to vary with the identity and the location of the mutated nucleotide. Among them, positional effects were the most evident. In relative to the modest effects of the PAM-distal mutations, profound effects were revealed for the PAM-proximal mutations. This indicates that PAM-distal mutations are much tolerated than PAM-proximal mutations^7,12,13^.

### Characterization of a highly sensitive “core” sequence

**Figure 3:**
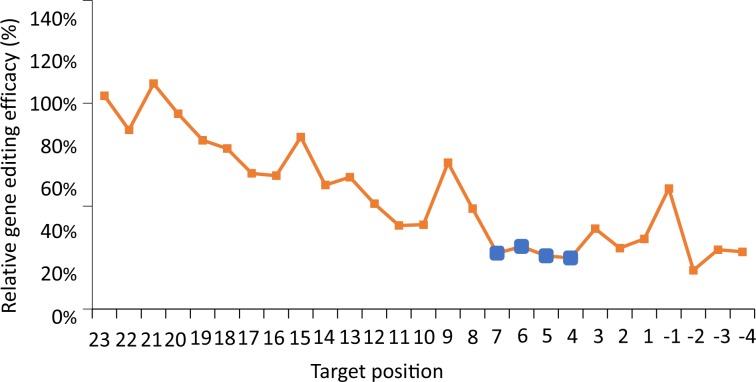
The tolerance profile of CRISPR/Cpf1 system. The tolerance profile is obtained by averaging the relative gene editing efficacy of all target mutations at each position.

Despite the varying editing efficacies across the target site, the most evident influence was revealed within a 4-bp “core” sequence, located from position (+4) to position (+7) downstream of PAM (Fig. 3). Similar to that of CRISPR/Cas9, gene editing was abolished by most of the mutations made at the “core” sequence. Also similar to CRISPR/Cas9, this ‘core’ sequence was located within a positively charged central channel of Cpf1 endonuclease^14^. Therefore for both CRISPR/Cas9 and CRISPR/Cpf1, a common “core” sequence was demonstrated to serve as a major determinant of targeting specificity.

In addition to the positional effects, varying editing efficacies were also revealed for the mutations made at the same target position. For example at position (+15) of *ALKBH5* target, rU:dG mismatch was the most tolerated, while rU:dT or rU:dC mismatches led to a sharp decrease in editing efficacy (Fig. 2C).

**Figure 4:**
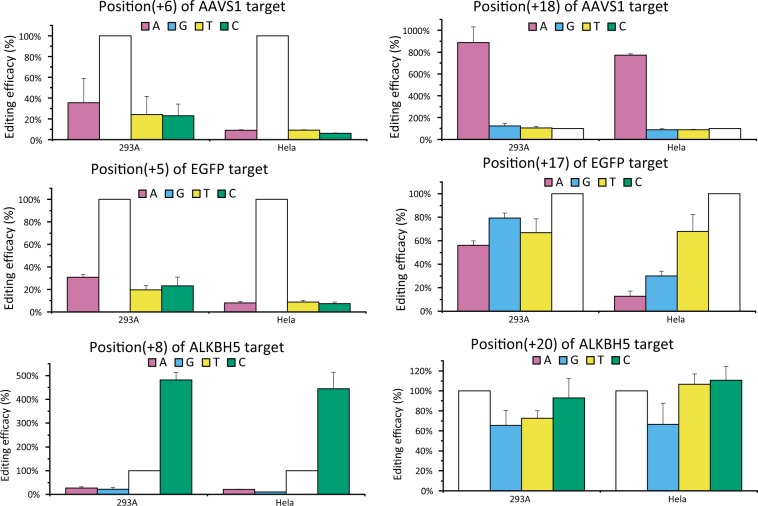
Comparative gene editing profiles of CRISPR/Cpf1 system in HEK293 and Hela cells.

In addition to the molecular components, host cells may also contribute to the gene editing as a background system. To address their potential influence, gene editing of a few targets was examined parallel with both HEK293 and Hela cells (Fig. 4). This led to comparable editing profiles. With *EGFP* target as an example, target mutations were barely tolerated at position (+5) within the “core” sequence, while mutations at position (+17) caused less compromised editing efficacies. It is of interesting to observe a greatly enhanced off-target gene editing at position (+18) of *AAVS1* target and position (+8) of *ALKBH5* target, for both HEK293 and Hela cells.

### Characterization of a ‘super’ off-target gene editing

In the assays, greatly enhanced off-target editing was revealed at several target positions. For *AAVS1* target, when nucleotide at position(+18) was mutated to adenine, gene editing efficacies were increased for an 8.8-fold. The effect was however not observed at position(+18) of the other targets, rendering it a sequence-dependent event. Similarly for *ALKBH5* target, rA:dC mismatch at position(+8) led to greatly enhanced off-target editing (4.8-fold). Taken with other sporadically reported cases^7,15,16^, a ‘super’ off-target gene editing was uncovered.

To further explore this phenomenon, orthogonal mutations were made with the deoxyribonucleotide of target DNA (dN) and the ribonucleotide of crRNA (rN). Reporter activation assays were performed with different combinations of mutated crRNA and target DNA. In addition to confirming the enhanced off-target gene editing, results showed that occurring of this event was determined by the identity of target nucleotide, rather than the nucleotides of crRNA. For position (+18) of *AAVS1* target, enhanced gene editing was observed when target nucleotide was dA, regardless of the identity of crRNA nucleotide. Similarly, for position(+8) of *ALKBH5* target, enhanced editing was observed when target nucleotide was dA, regardless of the identity of rN.

**Figure 5:**
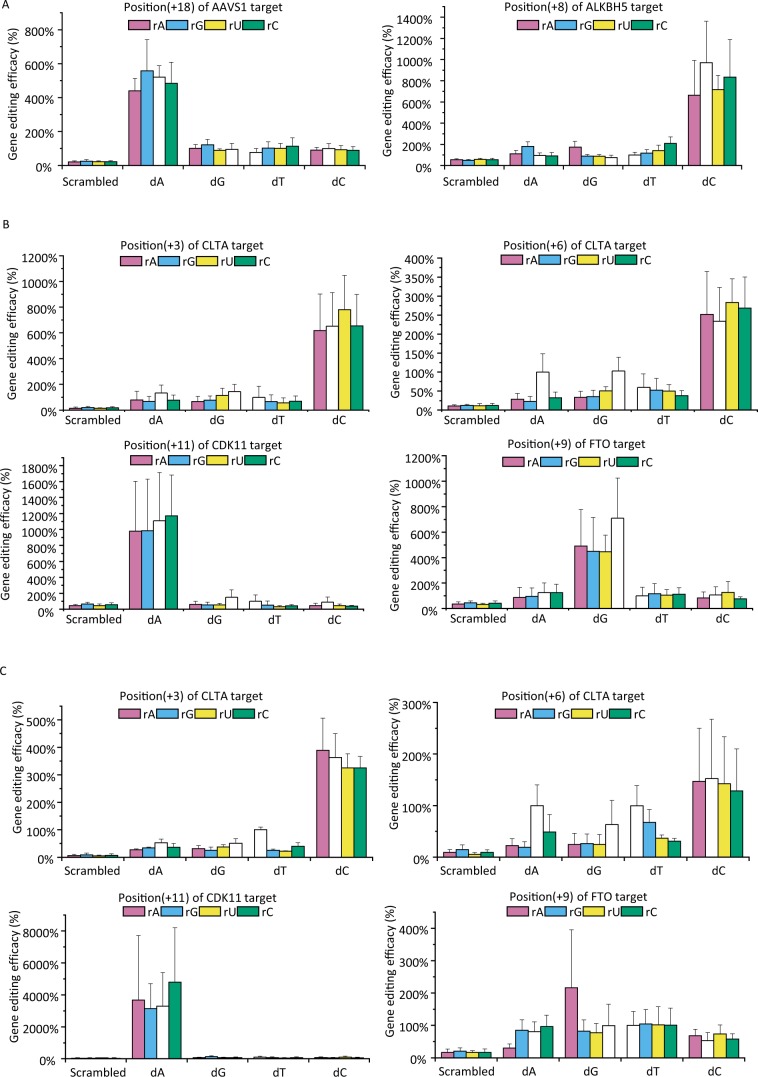
Gene editing efficacy of orthogonally mutated target nucleotide and crRNA nucleotide. (**A**) Relative gene editing efficacy of CRISPR/Cpf1 at position (+18) of *AAVS1* target, and position (+8) of *ALKBH5* target. (**B**) Relative gene editing efficacy of CRISPR/Cas9 at position (+3) and position (+6) of *AAVS1* target, position (+11) of CDK11 target, and position (+9) of FTO target. (**C**) Relative gene editing efficacy of CRISPR/Cas9(K855A) at position (+3) and position (+6) of CLTA target, position (+11) of CDK11 target, and position(+9) of FTO target.

We then examined our previous data with CRISPR/Cas9^7^, and identified enhanced off-target gene editing at a few target positions. Orthogonal assays performed with 4 target sequences showed that, the enhanced off-target gene editing was determined by the target nucleotide, rather than the nucleotide of crRNA (Fig. 5B). At position (+11) of CDK11 target, when target nucleotide is dA, off-target gene editing was increased for more than 8-fold. Taken these data together, a kind of ‘super’ off-target gene editing was characterized with CRISPR systems, representing a universal property of this widely applied gene editing platform.

### Strategy to optimize targeting specificity of CRISPR systems

To the mechanism of this phenomenon, we speculated that target nucleotides may form a certain interaction with protein domains of the endonuclease, and thereby contribute to targeting specificity in on-target and off-target gene editing. To explore this possibility, a sequence-modified Cas9 protein was examined with the reporter activation assay. The modified Cas9 carries a K855A point mutation and was shown to have improved target specificity^17^. As shown in Fig. 5C, the most profound effects were found with FTO target, in which the enhanced gene editing was almost abolished. While for CLTA and CDK11 targets, the off-target editing was largely maintained, with decreased levels (Fig. 5C). For example at position (+3) of CLTA target, the efficacies of off-target gene editing decreased from 600% to ~400%.

Therefore, despite the general increase in targeting specificity, a position-specific improvement was observed with the sequence-modified Cas9. This, on the one hand, lends support to the involvement of specific interaction between target nucleotide and protein domains; on the other hand, it proposes that by optimizing protein sequence and structure, targeting specificity of CRISPR system may be further improved.

## Discussion

Although strict targeting specificity is desirable for gene therapy, the systems we borrow from nature however exhibit a prominent property of redundancy, which is definitely of benefit to their hosts. For CRISPR systems borrowed from bacteria and archaea, targeting redundancy enables them to defend a batch of relevant virus species, instead of targeting only the virus from which the crRNAs are derived. Therefore, tolerance to certain off-target effects is theoretically preferred, as this may valuably extend their target ranges. In the present study, CRISPR/Cpf1 system was shown to have a similar specificity profile as that of CRISPR/Cas9, tolerating point mutation at most target positions. Also similar to that of CRISPR/Cas9, a 4 bp-long “core” sequence was identified to be the major determinant of targeting specificity in CRISPR/Cpf1.

In addition to the specificity profile, a ‘super’ off-target gene editing was surprisingly revealed for both CRISPR/Cpf1 and CRISPR/Cas9, depending on the specific target sequences. Target mutation at certain positions led to greatly enhanced gene editing efficacy, up to as high as 10-fold of that of the fully-matched target. When the effects were examined in terms of mutation position, no clear rule could be drawn. This suggests that sequence-independent factors, such as methylation level, GC content, surrounding chromatin content, nucleosome location, etc. may be involved in the phenomenon.

Besides our study, gene editing of single mutated crRNA/target sites has been previously investigated^8,9,15,16^. In their studies, sequence- and position-dependent tolerance profiles have been reported. To the aspect of enhanced ‘off-target’ effect, it was observed with some target mutation, but not reported for the crRNA mutation.

To explore the mechanism underlying the ‘super’ off-target gene editing, we speculated that target nucleotides may form certain interactions with protein domains of Cpf1, and thereby contribute to targeting specificity of the system. To this aspect, a sequence-modified Cas9 protein was tested with the reporter activation assay. Results showed that both the general and ‘super’ off-target gene editing got improved, in a position-specific manner. Although K855A mutation of Cas9 protein ameliorated off-target editing at positions +3, +6 and +11, the most profound effect was found with position +9. Identification of this position-specific improvement supports the involvement of specific interaction between target nucleotide and protein domains.

Targeting specificity is a long-term focus of our group. In previous studies performed with RNA interference (RNAi)^18–20^, a reporter-based assay was performed to examine the specificity of gene silencing. Although position-specific, rather than sequence-specific profiles were revealed with both RNAi and CRISPR systems, the ‘super’ off-target editing has never been found with RNAi, showing it is unique to CRISPR systems. Considering their potential harm to gene therapy^21^, we believe that more attention needs to be paid to the off-targeting gene editing, in particular the ‘super’ off-target gene editing of CRISPR systems.

## Materials and Methods

### Oligonucleotides and plasmids

DNA oligonucleotides were synthesized by Sangon Biotech (Beijing, China) and Invitrogen. pY010 and pcDNA3.1-hAsCpf1 plasmids were from Dr. Feng Zhang (Addgene, #69982), pU6-As-crRNA plasmid was from Dr. Jin-Soo Kim (Addgene, #78956). pTarget vector was from Biomics Biotechnologies (Nantong, China).

### sgRNA activity assay

Cells were grown in DMEM medium supplemented with 10% FBS, 100 units/ml penicillin and 100 µg/ml streptomycin (Life Technologies, Gibco). The cells were seeded into 48-well plates at a density of ~2.5 × 10^4^ cells/well one day before transfection. pCpf1 vector (200 ng/well), pcrRNA vector (200 ng/well) and pTarget plasmid (30 ng/well) carrying a target site of tested sgRNA were co-transfected into the cells at approximately 70% confluence, together with pRL-TK reference vector (5 ng/well). Activities of *Firefly* and *Renilla* luciferases were determined by a luminometer (Synergy HT, BioTek, USA). For each well, the activity of *Firefly* luciferase was normalized to that of *Renilla* luciferase. By comparing it with a sample without crRNA treatment, the editing efficacy of a crRNA was calculated. All the assays were performed in triplicate and repeated for at least three times.

### Statistics analysis

Analyses were performed with GraphPad Prism5 software. Data are presented as mean ±SD. One-way ANOVA analysis was used to evaluate the statistical significance. The significance level was set at p < 0.05.
